# CircRNA-mediated regulation of immune checkpoints in lymphoma: a multidimensional network perspective

**DOI:** 10.3389/fimmu.2026.1877435

**Published:** 2026-07-22

**Authors:** Jingjing Liu, Tianhua Zhao, Yanning Wu, Li Li, Wenjing Li

**Affiliations:** Hematology Department, Bethune International Peace Hospital, Shijiazhuang, Hebei, China

**Keywords:** circRNA, exosome, immune checkpoint, immunotherapy resistance, lymphoma, network biology, PD-L1, tumor microenvironment

## Abstract

Immune checkpoint inhibitors (ICIs) have revolutionized lymphoma treatment, yet resistance driven by complex regulatory networks remains a major hurdle. Circular RNAs (circRNAs) have emerged as central signaling hubs that integrate metabolic, inflammatory, and oncogenic cues to fine-tune immune checkpoints such as PD-L1 and CD47 in lymphoma. Here, we systematically dissect the molecular mechanisms by which circRNAs govern immune checkpoints in lymphoma, including nuclear transcriptional control, interactions with RNA-binding proteins (RBPs), competitive endogenous RNA (ceRNA) networks, and micropeptide translation. We differentiate between cell-extrinsic, exosome-mediated reprogramming of the tumor microenvironment and cell-intrinsic circRNA circuits within lymphoma cells spatially. Subtype-specific investigations demonstrate the importance of Epstein-Barr virus (EBV)-encoded circRNAs in immune evasion and the synergistic interactions between 9p24.1 amplification and circRNAs in classical Hodgkin lymphoma (cHL). While therapeutic approaches including antisense oligonucleotides, CRISPR-Cas13, and nanodelivery technologies demonstrate preclinical synergy with ICIs, circulating circRNAs show potential as dynamic indicators for predicting ICI responses. We also critically examine ongoing discussions about the flaws of current model systems, the technological constraints of current validation techniques, and the physiological significance of the ceRNA hypothesis. Positioning circRNAs as multimodal regulatory hubs, this review provides a theoretical framework for developing circRNA-based immunotherapies to overcome resistance in lymphoma.

## 1 Introduction

Lymphoma is a highly heterogeneous haematological malignancy where tumor-immune interactions dictate clinical outcomes (1, 2). The development of immune checkpoint inhibitors (ICIs), particularly those that target the Programmed Cell Death Protein 1 (PD-1)/Programmed Cell Death Ligand 1 (PD-L1) axis, has transformed the therapy landscape for classical Hodgkin lymphoma (cHL) and certain kinds of non-Hodgkin lymphoma. However, clinical success varies significantly, some patients obtain long-term full remission, while others undergo rapid disease progression. This diversity demonstrates that our understanding of the immune checkpoint regulation network remains insufficient (3–7). The development of drug resistance is not caused by the failure of a single pathway, but rather results from the interplay of multiple mechanisms. These include changes occurring within the tumor cells themselves—such as the loss of antigen-presenting function and the constitutive activation of oncogenic pathways—as well as external factors, such as immune components including suppressor myeloid cells and regulatory T cells, and the dynamic adaptive processes of the tumor microenvironment (TME) in response to therapeutic pressure (8, 9). The crux of the issue is the dysregulation of immune checkpoint molecule including PD-L1, CD47, LAG-3, and TIM-3. These molecules are not static biomarkers, but embedded in a densely interconnected network, with each performing a critical role as a key node inside it (10). We argue that circRNAs, as network hubs, provide an unexploited opportunity to rewire these dysregulated checkpoints.

Circular RNA (circRNA) was once regarded as a by-product of the splicing process, but has now emerged as key regulatory non-coding RNAs. Their covalently closed loop structure confers high stability, enabling it to persist both within and outside the cell (11, 12). Generated through reverse splicing, circRNAs can act as miRNA sponges, interact with RNA-binding proteins (RBPs), serving as scaffolds for signaling complexes, and, in some cases, encode functional micropeptides (13–16). The expression of circRNAs exhibits tissue- and cell-type-specificity, in the development and progression of cancer, they are extensively involved in various aspects of cellular proliferation, metastasis, metabolic reprogramming and immune regulation (17, 18).

CircRNAs do not act alone, rather, they serve as a central hub for signal integration, capable of concentrating oncogenic, inflammatory, and metabolic signals onto themselves and then translating these signals into the precise expression required by immunological checkpoints (19–21). CircRNAs’ integrative capacity allows them to build a strong link between the intrinsic properties of tumors and the immunological milieu, serving as a crucial hub. This review was primarily built on three principles: the principle of multidimensional regulation, which includes transcriptional, post-transcriptional, and post-translational levels, the principle of spatial compartmentalization, which distinguishes between intracellular and extracellular mechanisms, and the principle of context-dependence, which requires consideration of different lymphoma subtypes, genetic backgrounds, and the specific composition. This framework allows us to move beyond linear pathway models and propose a holistic, network-based view of circRNAs’ function. Throughout this review, we distinguish between mechanistically validated findings and theoretical models, and we highlight where quantitative data are lacking. By establishing this approach, we want to highlight the networked role of circRNAs in immune modulation.

Unlike previous reviews that catalog circRNA–checkpoint associations, we here (i) critically evaluate the physiological relevance of the ceRNA hypothesis, (ii) propose subtype- and space-specific intervention strategies, and (iii) highlight prognostic circRNA signatures derived from published literature.

## 2 Key checkpoints in the lymphoma immune microenvironment: network nodes and regulatory hierarchies

### 2.1 The PD-1/PD-L1 axis: a paradigm of dynamic regulation

In lymphoma research, the PD-1 and PD-L1 immune checkpoint pair has been the most extensively studied and is the best characterized (22, 23). In cHL, amplification occurs at the 9p24.1 locus, this amplification drives PD-L1 expression, resulting in its constitutive overexpression (24, 25). In diffuse large B-cell lymphoma (DLBCL), PD-L1 expression is primarily concentrated in the non-GCB subtype, and there is a clear association between the level of this expression and poor patient prognosis (26). It is important to clarify that PD-L1 expression is not a simple on-off binary state, rather, it is the result of a dynamic equilibrium. This equilibrium is determined by a combination of factors, including the activation status of oncogenic pathways (such as the NF-κB, STAT3 and MAPK pathways), the intensity of inflammatory signals (such as IFN-γ), and fluctuations in cellular metabolic states (such as hypoxic environments and changes in lipid metabolism) (27–33). This equilibrium is achieved at multiple levels. Some regulatory mechanisms occur at the transcriptional level, mediated by transcription factors such as STAT3 and NF-κB, others occur at the post-transcriptional level, involving molecules such as miR-138-5p, miR-570-3p and the RNA-binding protein HuR, while others occur at the post-translational level, exerting their effects through modification processes such as ubiquitination and glycosylation (34–37). It is precisely the existence of this multi-level regulatory mechanism that provides a natural platform for circRNAs to perform their integrative functions, capable of converging signals from various levels. In diffuse large B-cell lymphoma (DLBCL), circPCBP2 (hsa_circ_0026652) has been shown to promote PD-L1 expression through METTL3-mediated m6A modification and subsequent activation of the USP22/β-catenin axis, thereby suppressing CD8^+^ T cell activity and facilitating immune evasion (38) (**Table 1**).

**Table 1:** Multi-level regulation of PD-L1 expression in lymphoma.

Regulatory level	Key molecules/mechanisms	Examples in lymphoma/relevant context	Selected references
Transcriptional	Transcription factors (NF-κB, STAT3, MYC); 9p24.1 amplification	Constitutive PD-L1 overexpression in cHL due to 9p24.1 amplification; NF-κB and STAT3 activation in DLBCL	(24, 28)
Post-transcriptional	miRNAs (miR-138-5p, miR-570-3p); RNA-binding proteins (HuR); ceRNA networks	circPCBP2 sponges miR-33a/b to upregulate PD-L1 in DLBCL; HuR stabilizes PD-L1 mRNA	(36, 39)
Post-translational	Ubiquitination, glycosylation, phosphorylation	Glycosylation of PD-L1 stabilizes the protein and suppresses T-cell activity; ubiquitination regulates its degradation	(27, 37)

### 2.2 CD47: beyond the “don’t eat me” signal

The CD47 molecule could signal to macrophages via SIRPα to tell them “Do not eat me”, it is a classic example of a natural immune checkpoint (40–42). In a variety of hematologic malignancies, CD47 expression levels are upregulated, and this overexpression is strongly associated with a poor prognosis in patients (43–45). In addition to performing this classic signaling function, CD47 is also capable of driving tumor metastasis via a RhoA-dependent pathway (46). Current research into the regulatory mechanisms of CD47 has identified the involvement of transcription factors such as MYC and NF-κB, as well as the regulatory role of deubiquitinating enzymes (47, 48). However, direct circRNA-mediated regulation of CD47 in lymphoma has not yet been reported. Whether circRNAs modulate CD47 expression in lymphoma, either through miRNA sponging, RBP interactions, or other mechanisms, remains an open question and represents a priority for future investigation.

### 2.3 Emerging checkpoints: LAG-3, TIM-3, and co-regulatory networks

LAG-3 and TIM-3 are co-inhibitory receptors, these two molecules are frequently co-expressed on the surface of exhausted T cells. The expression levels of their ligands, MHC-II and galectin-9, are upregulated in the tumor microenvironment, and together they form a synergistic line of defense against immune activation (10, 49–52). The regulation of these checkpoints is often mediated through shared signaling pathways (such as NF-κB and STAT3) and shared transcriptional programs, thereby forming a functionally modular network. Given their ability to integrate multiple signals, circRNAs theoretically have the potential to regulate multiple checkpoints simultaneously. Because circRNAs can simultaneously target multiple mRNAs through shared miRNAs or RBPs, it is plausible that they may co-regulate interconnected checkpoint networks such as LAG-3 and TIM-3. However, direct evidence supporting this view in the field of lymphoma remains limited at present, and this hypothesis requires further experimental validation. Notably, in DLBCL, circSPEF2 has been shown to increase CD4^+^ T cell proportion and decrease regulatory T (Treg) cells through the miR-16-5p/BACH2 axis, suggesting its potential role in modulating the tumor immune microenvironment (53).

### 2.4 From linear pathways to networks: a conceptual leap

Viewing the regulation of immune checkpoints as a network rather than a simple linear pathway is a prerequisite for understanding the function of circRNAs. Within this network, transcription factors, microRNAs, RNA-binding proteins and circRNAs all constitute nodes, they are interconnected via regulatory edges, forming both feedback and feedforward loops, thereby endowing the entire system with the capacity for dynamic adaptation (54, 55). CircRNAs occupy a unique position within this network, their biogenesis is regulated by RNA-binding proteins and splicing factors, while they themselves can regulate other nodes through sponging, scaffolding or coding functions, thereby acting as hub nodes. Identifying these hub nodes is of great significance for the discovery of therapeutic vulnerabilities.

## 3 Molecular mechanisms: how circRNAs reshape the immune checkpoint network

### 3.1 The ceRNA paradigm: stoichiometry, redundancy, and network buffering

According to the ceRNA theory, circRNAs can compete with messenger RNAs for shared microRNA binding sites, effectively sequestering microRNAs (39, 56). The ceRNA hypothesis has been widely invoked, but its physiological relevance faces stoichiometric challenges. Nevertheless, a subset of highly expressed circRNAs, such as circPCBP2 in DLBCL, can reach sufficient abundance to sponge miRNAs and upregulate PD-L1. For the majority of circRNAs, however, functional validation is required. Functional redundancy is a deeper problem. Since several circRNAs frequently act as a “sponge” to support a single microRNA, the effects of knocking down one circRNA may be offset by the functions of other members. This explains why only minor phenotypic changes are seen after the knockdown of specific circRNAs. From a network perspective, we propose that circRNAs may function as a collective buffering system, preserving the activity of microRNAs at a comparatively steady level through cooperative interactions. However, this hypothesis awaits experimental validation through quantitative measurements of circRNAs and miRNA copy numbers in defined subcellular compartments. This realization has broad implications for therapeutic approaches: targeting a single circRNA could not be enough to provide a meaningful therapeutic effect, instead, it might be essential to target several circRNAs at once or their upstream shared regulatory mechanisms. Subcellular localization adds further complexity. While miRNAs are predominantly cytoplasmic, many circRNAs reside in the nucleus. Even when co-localized, their relative concentrations and spatial distribution may affect the efficiency of competition. To accurately ascertain the characteristics of these interactions between circRNAs and microRNAs, future study will need to employ methods like quantitative single-molecule imaging, subcellular separation, and gene editing. In DLBCL, circPCBP2 acts as a sponge for miR-33a and miR-33b, thereby lifting the inhibitory effect of these microRNAs on PD-L1, while also promoting stem-like characteristics and chemoresistance in tumor cells (57). Following knockdown of circPCBP2, the activity of miR-33a and miR-33b was restored, PD-L1 expression levels subsequently decreased, and therapeutic efficacy was enhanced, these findings validate the potential therapeutic value of this regulatory axis. Similarly, in DLBCL, circ_0003645 functions as a miR-335-5p sponge to upregulate nuclear factor I/B (NFIB), thereby promoting cell proliferation and glycolysis (58). Although there is currently insufficient evidence to confirm whether a direct regulatory relationship exists between circRNAs and CD47, it has been found that CDR1as (also known as ciRS-7) can sequester miR-7, which can regulate CD47 expression (13, 59). Future research could involve systematic screening in lymphoma to identify circRNAs that interact with microRNAs targeting CD47, this may represent a promising avenue of investigation. Bioinformatics methods have already been used to construct ceRNA networks associated with lymphoma and to identify circRNA features correlated with immune infiltration status and clinical outcomes (60, 61). However, these findings still need to be validated through experimental approaches such as dual-luciferase assays, RNA immunoprecipitation (RIP) and CRISPR gene editing, in order to distinguish between circRNAs that play a genuine driving role and those that act merely as passengers (62).

More critically, the physiological relevance of the ceRNA hypothesis has long been challenged from a quantitative perspective (63). The absolute copy number of most circRNAs is far lower than that of their presumed target miRNAs, and the number of binding sites provided by a single circRNA molecule is usually insufficient to significantly alter the effective concentration of miRNAs (64). For example, in a typical mammalian cell, a circRNA with median abundance (approximately 10–50 copies per cell) can sequester a negligible number of miRNA molecules relative to the thousands to tens of thousands of copies of miRNAs present intracellularly (65). Hence, a single circRNA–miRNA pairing is unlikely to produce a functional competitive effect. However, this does not invalidate the ceRNA mechanism; rather, it calls for a renewed view of its mode of action. On one hand, a small number of highly abundant circRNAs (such as circPCBP2 in DLBCL, which reaches several hundred copies per cell) do possess sufficient “sponge” capacity (57). On the other hand, multiple low-abundance circRNAs can act cooperatively to form a collective buffering network that maintains miRNA activity within a steady-state range (66). Moreover, competitive binding does not always require titration of the entire miRNA pool — in specific subcellular compartments (e.g., P-bodies or exosome-biogenesis zones), the local concentrations of circRNAs and miRNAs may be an order of magnitude higher than global averages (67). Future studies should quantitatively report absolute copy numbers of both circRNAs and miRNAs, their subcellular localization ratios, and target occupancy rates, and combine single-molecule imaging with gene-editing technologies to truly resolve the physiological contribution of the ceRNA hypothesis.

### 3.2 circRNA-RBP interactions: from binary classification to dynamic scaffolds

Through sequence-specific motifs, circRNAs can interact with RNA-binding proteins (RBPs), either as scaffolds to organize protein complexes or as sponges to sequester RBPs (68, 69). This binary classification, nevertheless, might be too simplistic. A single circRNA can interact with several RBPs at once, and when biological circumstances change, its functional status changes dynamically. For example, it might serve as a scaffold in normal circumstances but as a sponge in stressful situations. Although traditional steady-state tests frequently fail to capture such dynamic changes, this functional flexibility provides an essential mechanism for cells to achieve adaptive control.

RBPs are intrinsically multifunctional and can form complexes. Many RBPs have numerous RNA-binding domains, allowing them to bind to multiple targets simultaneously. When circRNA interacts with RBPs, a ribonucleoprotein complex is created. In this scenario, the circRNA serves as a scaffold, bringing together several RBPs and messenger RNAs to form a local regulatory center. CircRNAs’ role is elevated from this perspective, it is no longer just a passive sponge, but also an active organizer.

In terms of therapeutic potential, many RBPs possess structured RNA-binding pockets, which provide targets for the development of small-molecule drugs. Competitive peptides or small molecules can be designed to target conserved sequence motifs present in the interaction regions between circRNAs and RBPs, however, the current lack of high-resolution structural information constitutes a major technical obstacle. In lung cancer models, circRNAs have been shown to accelerate the degradation of PD-L1 by competing with HuR for binding sites, or indirectly enhance HuR activity by acting as a sponge to adsorb microRNAs that target HuR (70). Whether similar mechanisms operate in lymphoma remains to be investigated. Recent advances in crosslinking immunoprecipitation (CLIP) have begun to map circRNA-RBP networks in immune cells. Beyond RBP interactions, circRNAs can also modulate signaling pathways through miRNA sponging. In T-ALL, circ-PRKDC sponges miR-653-5p to upregulate Reelin (RELN) and activate PI3K/AKT/mTOR signaling (71), demonstrating how circRNA-mediated ceRNA networks can intersect with major oncogenic signaling cascades.

### 3.3 circRNA-encoded micropeptides: therapeutic opportunities in the cryptic proteome

Some circRNAs can convert themselves into functional peptides via internal ribosome entry sites (IRES) or m6A modification (16, 72). This discovery challenges the long-held view that non-coding RNAs are incapable of encoding proteins, revealing an additional layer of proteomic complexity. However, the extent to which such translation occurs is uncertain. Early reporter gene experiments or overexpression models may have overstated the frequency of translation, as ribosomal profile analyses show that only a small proportion of circRNAs bind to ribosomes.

The conditions that cause translation should also be considered. Stressful situations (such as hypoxia, viral infection, or chemotherapy) may stimulate the translation of circRNAs by modifying m6A modifications or IRES activity, implying that micropeptides may function as stress regulators. By competing with full-length proteins for binding sites or by interfering with the post-translational modification of full-length proteins, many micropeptides have a “dominant-negative” effect in terms of functional mechanisms. As a result, they can have notable effects even at relatively low concentrations.

In melanoma, a 108-amino acid micropeptide encoded by circPIAS1 has been shown to inhibit STAT1 phosphorylation by enhancing the SUMOylation of STAT1, thereby blocking immunogenic ferroptosis and reducing the therapeutic efficacy of immune checkpoint inhibitors (73). The combination of antisense oligonucleotides (ASOs) targeting this micropeptide with PD-1 inhibitors can achieve synergistic inhibition of tumor growth, this finding provides proof of concept for relevant therapeutic strategies. In lymphoma, frequent chromosomal translocations and gene rearrangements may give rise to unique fusion circRNAs. It is hypothetical that the micropeptides encoded by these circRNAs could possess oncoprotein activity or serve as neoantigens. Although this concept is supported by proof-of-principle evidence from melanoma (73, 74), the systematic identification of circRNA-encoded micropeptides in lymphoma, and the elucidation of their role in immune evasion or antigenicity, represents an important future research direction that requires direct experimental investigation.

Of particular interest, virus-encoded circRNAs (v-circRNAs) such as those from EBV and their translation products represent a promising but still exploratory direction. It is hypothesized that v-circRNA-derived micropeptides may provide unique immunotherapeutic targets and sources of tumor-specific antigens in lymphoma, given their sequence heterogeneity and potential for high HLA class I binding affinity (75). However, direct evidence for micropeptide translation from v-circRNAs in lymphoma and their immunogenicity in patients remains to be established. This represents a priority area for future investigation combining ribosome profiling, mass spectrometry, and T-cell functional assays. Viral-derived micropeptides are highly heterologous — they are neither fully identical to host proteins nor identical to classical viral antigens, but their sequences contain virus-specific amino acid motifs, conferring a natural advantage as neoepitopes. Computational predictions indicate that v-circRNA-derived micropeptides often rank highly in HLA class I binding affinity, suggesting they can be effectively recognized by CD8^+^T cells (75). Moreover, circRNA micropeptides can be exploited for clinical translation through three routes: (1) As tumor-specific neoantigens for personalized vaccines — predicted micropeptide sequences can be loaded onto dendritic cells or incorporated into mRNA/peptide vaccines to elicit anti-tumor T-cell responses (76). (2) As combinatorial targets for immune checkpoint therapy-designing ASOs or small-molecule inhibitors against circPIAS1-like micropeptides not only blocks their negative regulation of pathways such as STAT1 but also releases the immunogenic cell death signals that micropeptides have “hidden”. (3) As liquid biopsy and prognostic markers-because circRNAs are highly stable, their encoded micropeptides may also be released into the tumor microenvironment via MHC presentation; detection of autoantibodies against these micropeptides could reflect the anti-tumor immune status (77). For EBV-associated lymphoma, systematically identifying v-circRNA translation products and validating their immunogenicity holds promise for opening a precision immunotherapy direction of “circRNA-encoded neoantigens”. This field is still in its infancy and urgently requires integration of ribosome profiling, mass spectrometry, and T-cell functional assays to define the clinically meaningful micropeptide repertoire in lymphoma.

### 3.4 Nuclear circRNAs: covert regulation at the transcriptional level

Some circRNAs localize to the cell nucleus, where they regulate gene transcription (78, 79) These nuclear-localized circRNAs (including those formed by a combination of exons and introns) are capable of interacting with chromatin modification complexes, some even bind directly to promoter regions, forming RNA-DNA triplets or R-loop structures, thereby adding a new dimension to the regulation of immune checkpoint genes. This mechanism operates independently of post-transcriptional regulation, revealing that circRNAs are capable of exerting control over immune checkpoint genes at the earliest stages of gene expression. Intranuclear circRNAs have also been found to participate in the transcriptional regulation of oncogenes in acute myeloid leukemia (80), suggesting that such mechanisms may be widely present in hematological malignancies (**Table 2**).

**Table 2:** Major mechanistic studies of circRNA function (general cancer models, relevant to lymphoma).

Mechanistic category	Key circRNA/model	Findings relevant to immune checkpoints	Reference
ceRNA (miRNA sponge)	CDR1as (ciRS-7)	Efficiently sponges miR-7; miR-7 can target CD47, providing indirect link	(13, 14)
ceRNA (stoichiometry debate)	Quantitative analysis	Most circRNAs are low-abundance; casts doubt on physiological relevance of sponging	(58, 59, 61, 65)
RBP interaction/scaffold	circRNA/HuR	circRNAs can compete with HuR to destabilize PD-L1 mRNA or sequester RBPs	(36, 64)
Micropeptide translation	circPIAS1 (melanoma)	Encodes 108-aa peptide that enhances STAT1 SUMOylation, blocks immunogenic ferroptosis	(73)
Nuclear transcriptional regulation	circRNAs in AML	Nuclear circRNAs interact with chromatin modifiers; potential for PD-L1 transcriptional control	(77)
Exosome-mediated transfer	Various cancers	Exosomal circRNAs reprogram TME; selectively secreted and functionally active	(76, 77)

## 4 Spatial dimensions: cell-intrinsic and cell-extrinsic synergistic networks

The molecular mechanisms described above—ceRNA networks, RBP scaffolding, micropeptide translation, and nuclear regulation—do not operate in isolation. Their functional impact depends critically on the spatial context: whether they act within a single lymphoma cell (cell-intrinsic) or are transmitted across cells to reshape the broader tumor microenvironment (cell-extrinsic). Disentangling these two spatial dimensions is essential for understanding how circRNA networks achieve both local autonomy and global coordination.

### 4.1 Cell-intrinsic mechanisms: the cornerstone of autonomous regulation

The circPCBP2-miR-33a/b-PD-L1 axis exemplifies cell-intrinsic regulation. Within lymphoma cells, circPCBP2 autonomously driving PD-L1 expression while concurrently promoting stem-like properties and chemoresistance (57). The phenomena of immune evasion and treatment resistance converge at the level of single-stranded circRNA, making it a relatively ideal therapeutic target.

### 4.2 Cell-extrinsic mechanisms: exosome-mediated remote reprogramming

Tumor cells are capable of remotely remodeling the tumor microenvironment via the exosome pathway (81, 82). Whether exosomal circRNAs actually serve as a functional purpose remains a matter of debate. Does it serve as a functional signaling molecule, or is it merely cleared away by the cell as waste? Those who support the notion that it is functional cite several pieces of evidence to support their view, such as the selective secretion of this type of circRNA, its ability to persist stably within recipient cells, and its capacity to induce functional changes in those cells. Opponents, however, point out that the concentration of circRNA within exosomes is actually very low.

The debate on whether exosomal circRNAs are functional signaling molecules or merely cellular waste remains unresolved. On one hand, evidence supporting their functional relevance includes selective enrichment of certain circRNAs into exosomes, indicating active sorting rather than random packaging; their exceptional stability in body fluids and efficient uptake by recipient cells; and the cumulative effect of hundreds or thousands of exosomes internalized by a single recipient cell, which may reach a local concentration sufficient to initiate downstream signaling cascades. On the other hand, sceptics point out that the concentration of circRNAs within individual exosomes is extremely low, and it remains unclear whether the amount delivered to recipient cells is sufficient to exert meaningful biological effects under physiological conditions. In this context, we put forward the following perspective: exosomal circRNAs may act as triggers in signal amplification networks. Rather than completing the entire regulatory task by themselves, a small number of circRNA molecules delivered via exosomes could relieve miRNA-mediated inhibition inside recipient cells or cooperate with endogenous circRNAs to “unlock” non-linear amplification reaction networks, thereby producing a disproportionately large downstream effect. This “trigger” hypothesis is consistent with known principles of signal amplification in regulatory networks, but its physiological relevance in lymphoma requires direct experimental validation, such as blocking the secretion of specific circRNAs or using single-exosome resolution detection methods. For example, exosomal circRNAs can compete for key immunosuppressive miRNAs, causing a small upregulation of target mRNAs (such as IFN-γ or immune effector molecules) that are otherwise repressed in recipient cells (83). This initial perturbation is then dramatically amplified through transcription-factor positive-feedback loops, ultimately resulting in a significant immunological phenotypic shift. Thus, low abundance does not necessarily equate to low function. We propose that exosomal circRNAs may initiate signal amplification cascades by relieving miRNA-mediated repression in recipient cells, thereby producing disproportionately large downstream effects through intrinsic network amplification mechanisms. Future efforts should develop single-exosome resolution detection methods and use gene editing to block the secretion of specific circRNAs, in order to directly validate their causal role in lymphoma microenvironment remodeling.

The circRNA profiles carried by exosomes secreted by tumor cells and immune cells differ with respect to the heterogeneity of origin, and their functions occasionally even show opposing trends. This calls for the development of separation techniques capable of differentiating between cell origins. From a therapeutic standpoint, preventing tumor cells from secreting exosomes can stop immunosuppressive signals from traveling to far-off locations. On the other hand, we can deliver therapeutic circRNAs to target areas using modified exosomes, allowing for cell-type-specific therapies.

### 4.3 Integration of dual spaces: from local to global

**Figure 1:**
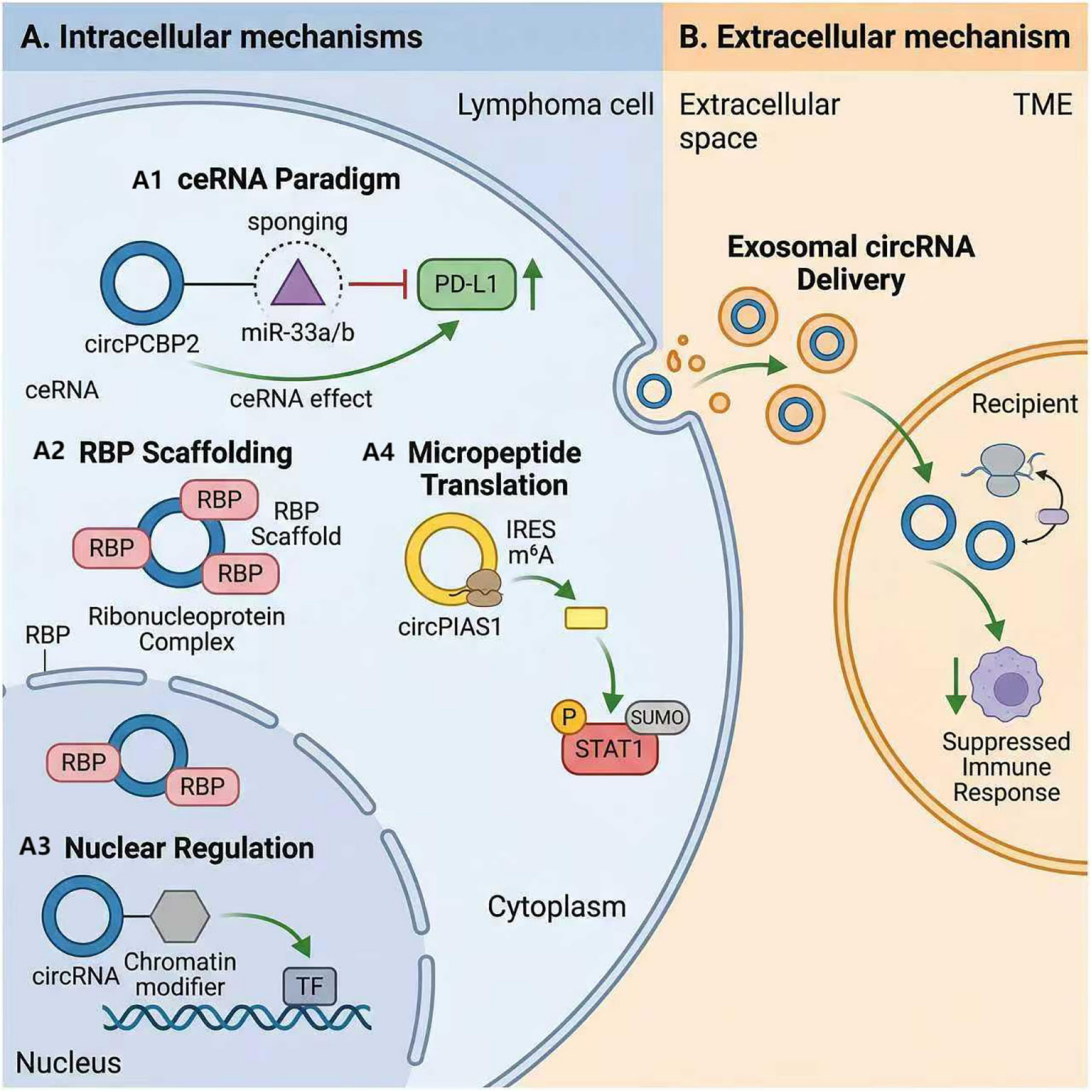
Multidimensional regulatory network of circRNAs on immune checkpoints in lymphoma. **(A1)** Intracellular mechanisms: (1) ceRNA paradigm: circPCBP2 sponges miR-33a/b to upregulate PD-L1. **(A2)** RBP scaffolding: circRNAs organize RNA-binding proteins into ribonucleoprotein complexes. **(A3)** Micropeptide translation: circPIAS1 encodes a peptide that modulates STAT1 SUMOylation/phosphorylation. **(A4)** Nuclear regulation: Nuclear-localized circRNAs interact with chromatin modifiers to control transcription. **(B)** Extracellular mechanism: Lymphoma cells secrete exosomes containing functional circRNAs, which reprogram recipient cells in the tumor microenvironment, promoting immunosuppression. Evidence level note: Mechanisms shown in panels **(A1, A3)** are validated in lymphoma; panels **(A2, A4)** include mechanisms extrapolated from other cancers or currently hypothetical. Solid lines represent mechanisms with direct evidence; dashed lines represent hypothetical or extrapolated mechanisms.

Immune checkpoint expression levels are set by intracellular circuits in lymphoma cells, while exosome-mediated circuits can alter the tumor microenvironment and exacerbate immune suppression. Combination therapy approaches that concurrently target both compartments have a theoretical foundation since the regulatory systems functioning across these two spatial dimensions work in concert to create a resilient system (**Figure 1**).

## 5 Subtype-specific circuits: integrating genetic background and viral factors

In cHL, amplification of the 9p24.1 locus drives constitutive PD-L1 expression (24), the presence of this protective mechanism may enhance the resistance of tumor cells to immune attack.

The circRNAs encoded by the EBV, such as circBARTs and other v-circRNAs, are able to bind to miR-203 via a sponge-like mechanism, thereby upregulating PD-L1 expression and ultimately promoting tumor immune evasion (84–86). These viral circRNAs have evolved to exploit the host’s microRNA network for immune evasion, effectively competing with host circRNAs for shared miRNA binding sites. Some viral circRNAs share overlapping microRNA binding profiles with the host’s own circRNAs, thereby creating a competitive relationship between the two. This may explain why the host’s non-coding RNA network undergoes remodeling following infection.

There are significant differences in circRNA expression profiles between the germinal center B-cell (GCB) and the non-germinal center B-cell (non-GCB) subtypes of diffuse large B-cell lymphoma (DLBCL). circPCBP2, whose functional role in PD-L1 regulation has been noted above, is significantly upregulated in the non-GCB subtype and correlates with PD-L1 expression and poor patient outcomes (57). Within the non-GCB subtype, high PD-L1 expression correlates with circPCBP2 upregulation, suggesting that circRNAs may act as a bridge linking genetic subtypes to immunophenotypes. Similarly, circ_OTUD7A is upregulated in DLBCL and promotes cell proliferation, migration, and invasion by sponging miR-431-5p to upregulate forkhead box P1 (FOXP1) (87), further supporting the oncogenic role of circRNAs in DLBCL pathogenesis. However, it remains unclear whether these differences in circRNA expression are drivers of disease onset and progression or merely epiphenomena.

**Figure 2:**
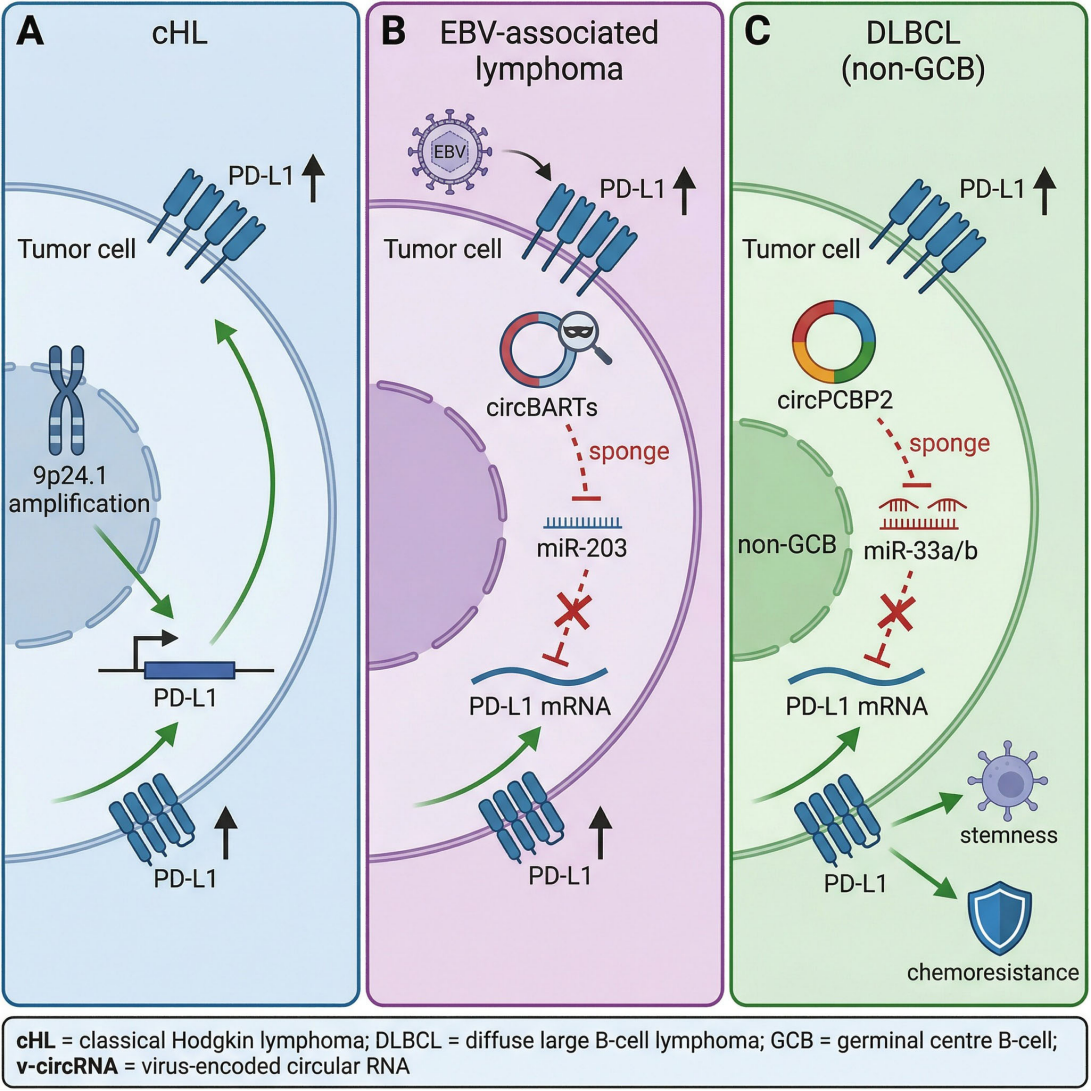
Subtype-specific circRNA circuits in lymphoma immunoediting. **(A)** In classical Hodgkin lymphoma, 9p24.1 amplification drives constitutive PD-L1 expression. **(B)** In EBV-associated lymphomas, viral circRNAs (e.g., circBARTs) sponge miR-203 to upregulate PD-L1, promoting immune evasion. **(C)** In diffuse large B-cell lymphoma, the non-germinal center B-cell subtype shows circPCBP2 upregulation, which sponges miR-33a/b to disinhibit PD-L1, promoting stemness and chemoresistance. Evidence level note: All three mechanisms shown are supported by direct experimental evidence in the indicated lymphoma subtypes. The dashed boxes indicate aspects of these mechanisms (e.g., precise molecular details or clinical relevance) that require further validation.

In mantle cell lymphoma (MCL) and natural killer T-cell lymphoma (NKTCL), subtype-specific circRNA expression has also been linked to prognosis and tumor proliferation (88, 89). For instance, in NKTCL, circKIF4A is significantly upregulated and correlates with poor prognosis through sponging miR-1231 to upregulate PDK1 and BCL11A, promoting glycolysis and tumor progression (90). In T-cell acute lymphoblastic leukemia (T-ALL), circ_0000745 promotes cell proliferation and inhibits apoptosis by sponging miR-193b-3p to upregulate NOTCH1 (91). Conversely, circ_0000094 functions as a tumor suppressor in T-ALL by sponging miR-223-3p to upregulate F-box and WD repeat domain containing 7 (FBW7) (92), highlighting the context-dependent and often opposing roles of circRNAs within the same malignancy. Consistent with the concept of context-dependent regulation, previous studies have shown that circRNA expression is highly altered in MCL (88) and that immune checkpoint genes such as PD-L1 and CD47 exhibit subtype-specific expression patterns in DLBCL, with significantly higher expression in the ABC subtype than in the GCB subtype (26, 57). These findings reinforce the central premise of this review: circRNA-mediated regulation is highly context-dependent and likely coordinates with immune checkpoint expression in a lymphoma-subtype-specific manner (**Figure 2**, **Table 3**).

**Table 3:** Key experimental evidence of circRNA-mediated immune checkpoint regulation in lymphoma.

circRNA/signature	Lymphoma subtype	Immune checkpoint/pathway	Experimental model	Level of evidence	Direct/indirect	Validation method	Clinical relevance	Reference
circPCBP2	DLBCL (non-GCB)	PD-L1 (via miR-33a/b)	Cell lines, xenograft	Validated in lymphoma	Direct	shRNA knockdown, luciferase, RIP	↑ stemness, chemoresistance, poor prognosis	(39)
circ_0026652	DLBCL	PD-L1 (via m6A/miR-1287-5p/USP22/β-catenin)	Cell lines, xenograft, patient samples	Validated in lymphoma	Direct	MeRIP, RIP, ChIP, co-culture	↑ immune evasion, ↓ CD8^+^ T cell activity	(38)
circADARB1	NKTCL	p-STAT3	Cell lines	Validated in lymphoma	Direct	qPCR, knockdown, rescue	↑ proliferation; potential biomarker	(89)
circKIF4A	NKTCL	PDK1/BCL11A (glycolysis)	Cell lines, patient cohorts	Validated in lymphoma	Direct	qPCR, RIP, luciferase, IHC	Predicts poor OS and PFS	(90)
circBARTs (viral)	EBV+ lymphomas	PD-L1 (via miR-203)	Cell lines, patient samples	Validated in lymphoma	Direct	Luciferase, overexpression	↑ immune evasion	(84)
circSCORE (4-circRNA panel)	MCL	N/A (prognostic)	Patient cohorts (MCL2, MCL3)	Validated in lymphoma	Direct	RNA-seq, LASSO, Cox regression	Predicts TTP and LSS	(83, 85)
circ_0003645	DLBCL	NFIB (glycolysis)	Cell lines	Validated in lymphoma	Direct	qPCR, RIP, luciferase, WB	↑ proliferation, glycolysis	(58)
circ_OTUD7A	DLBCL	FOXP1	Cell lines	Validated in lymphoma	Direct	qPCR, WB, Transwell, flow cytometry	↑ proliferation, migration, invasion	(87)
circSPEF2	DLBCL	miR-16-5p/BACH2 (Treg/CD4 balance)	Cell lines, co-culture	Validated in lymphoma	Direct	qPCR, RIP, luciferase, flow cytometry	Modulates tumor immune microenvironment	(53)
circ_0000745	T-ALL	NOTCH1	Cell lines	Validated in lymphoma	Direct	qPCR, RIP, luciferase, WB	↑ proliferation, ↓ apoptosis	(91)
circ_0000094	T-ALL	FBW7	Cell lines	Validated in lymphoma	Direct	qPCR, RIP, luciferase, WB	Tumor suppressor	(92)
circ-PRKDC	T-ALL	PI3K/AKT/mTOR (via miR-653-5p/RELN)	Cell lines	Validated in lymphoma	Direct	qPCR, RIP, luciferase, WB	↑ autophagy, apoptosis upon knockdown	(71)
circPIAS1-encoded peptide	(melanoma)*	STAT1/ferroptosis	Mouse models	Inferred from other cancers	Indirect	Ribosome profiling, CRISPR	Blocks immunogenic ferroptosis; ICI resistance	(73)
CDR1as (ciRS-7)	(breast, others)*	CD47 (via miR-7)	Cell lines	Inferred from other cancers	Indirect	Luciferase, sponge assays	Not yet studied in lymphoma	(13, 14, 59)
circNF1	(esophageal)*	PD-L1	Mouse models	Inferred from other cancers	Indirect	shRNA, IHC	Synergy with anti-PD-1 in solid tumors	(93)

*Explicitly noted: not yet validated in lymphoma; evidence derived from indicated cancer type(s).↑ means promotes, ↓ means suppresses.

### 5.1 Prognostic value of circRNAs in lymphoma

Beyond subtype-specific expression, circRNA signatures have shown prognostic potential. In MCL, a circRNA-based risk model (circSCORE) was developed in the MCL2 training cohort and validated in the independent MCL3 cohort, where it effectively identified younger patients at high risk of disease progression following cytarabine-containing therapy (88). A subsequent study further supported circSCORE’s prognostic value in the relapsed/refractory MCL setting (94). For DLBCL, high circPCBP2 expression has been linked to stemness, chemoresistance, and poor outcome, at least in part through PD-L1 upregulation (57). Other circRNAs (e.g., circAPC) have also been associated with aggressive disease and inferior survival (95, 96). Collectively, these findings suggest that circRNA-based classifiers could refine risk stratification and potentially guide immunotherapy decisions in lymphoma.

## 6 Clinical translation: from biomarkers to targeted therapies

The therapeutic applications discussed in this section are predominantly preclinical and experimental. With the exception of cell-based proof-of-concept studies, none of these approaches have advanced to clinical testing in lymphoma. CircRNAs exhibit exceptional stability in bodily fluids, a property that makes them an ideal class of liquid biopsy biomarkers (97). CircRNAs encapsulated within exosomes may carry even greater informational value, as they reflect a process of active cellular secretion.

The molecular foundation for the precise targeting of circRNAs is provided by the existence of the back-splicing junction (BSJ) (98). Certain circRNAs can be specifically degraded by antisense oligonucleotides (ASOs) that target BSJ. Advances in lipid nanoparticle (LNP) technology are quickly addressing delivery-related challenges (99, 100). Precise, cell-specific administration could potentially be achieved by functionalizing LNP with targeting ligands such anti-CD20 antibodies, although this approach remains experimental and has not yet been validated in lymphoma (101) (**Table 4**).

**Table 4:** Emerging therapeutic and translational strategies targeting circRNAs.

Strategy	Technology/approach	Application in cancer (preclinical)	Advantage/synergy	Key reference
circRNA degradation	ASOs targeting back-splicing junction (BSJ)	Specific knockdown of oncogenic circRNAs	High specificity; can combine with ICIs	(90)
circRNA degradation	CRISPR-Cas13d	Outperforms shRNA in identifying functional circRNAs	Efficient and programmable	(91)
Delivery system	Lipid nanoparticles (LNPs) with targeting ligands (e.g., anti-CD20)	Cell-type-specific delivery of circRNA-targeting ASOs	Overcomes systemic toxicity; enables precision therapy	(91, 92)
circRNA as vaccine	Engineered circRNAs encoding tumor antigens	circRNA cancer vaccines drive immunity in hard-to-treat malignancies	Stable, prolonged antigen expression	(99, 100)
Synergistic combination	circRNA inhibitor + anti-PD-1/PD-L1	In solid tumors (e.g., circNF1 + anti-PD-1)	Lowers T-cell activation threshold; prevents compensatory resistance	(92, 94)

**Figure 3:**
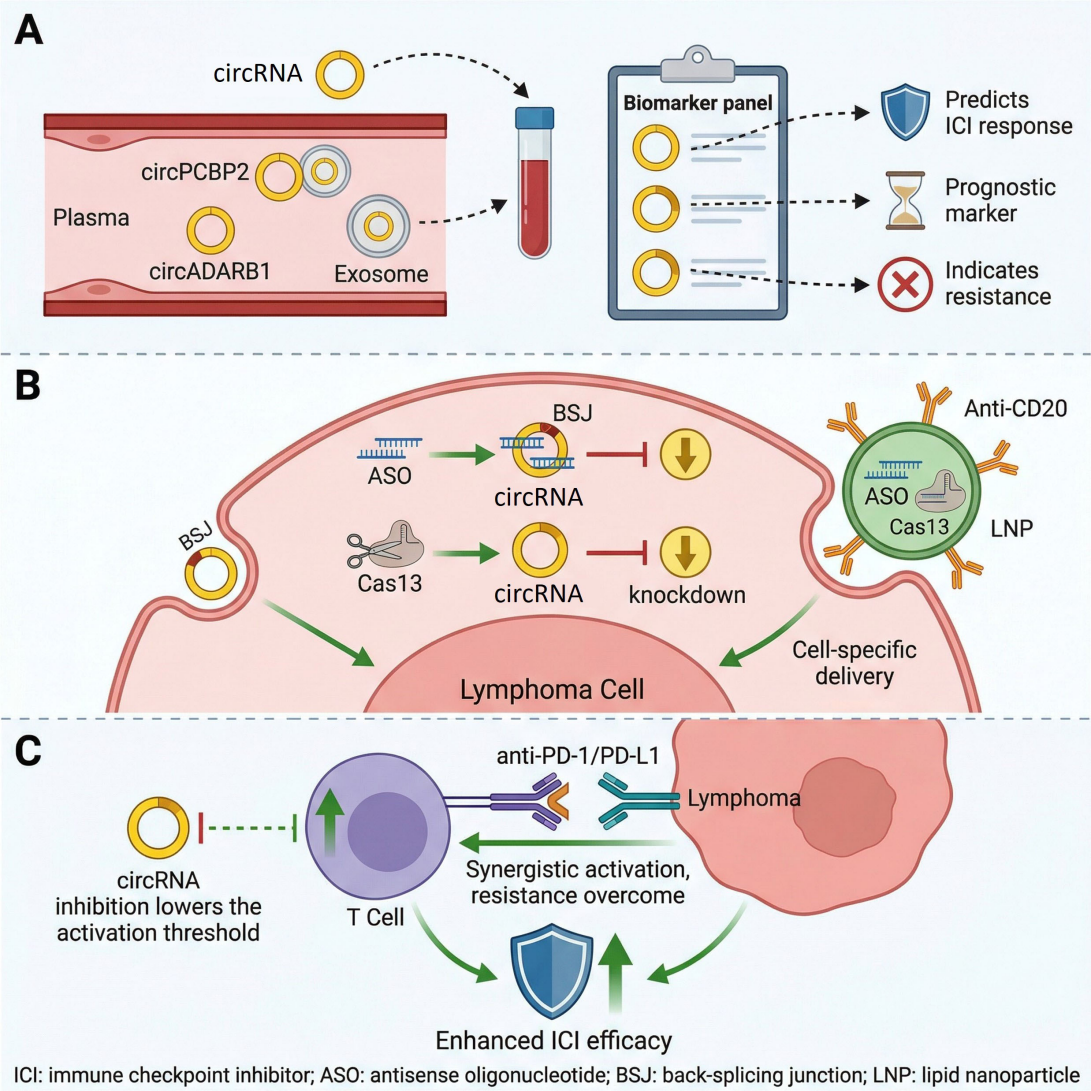
Clinical translation landscape of circRNAs in lymphoma. **(A)** Biomarker applications. CircRNAs (e.g., circPCBP2, circADARB1) detected in liquid biopsies (blood/plasma) or exosomes serve as dynamic functional biomarkers for predicting ICI response, prognosis, and resistance. **(B)** Therapeutic strategies. Multiple approaches target oncogenic circRNAs: (1) Antisense oligonucleotides targeting back-splicing junctions for specific degradation; (2) CRISPR-Cas13 systems for RNA knockdown; (3) Lipid nanoparticles functionalized with targeting ligands (e.g., anti-CD20) for cell-specific delivery. **(C)** Synergistic combinations. circRNA inhibition lowers the threshold for T-cell activation and synergizes with anti-PD-1/PD-L1 antibodies to overcome resistance. Abbreviations: ICI, immune checkpoint inhibitor; ASO, antisense oligonucleotide; BSJ, back-splicing junction; LNP, lipid nanoparticle. Evidence level note: The biomarker applications **(A)** are supported by clinical-scale studies, though prospective validation is ongoing. The therapeutic strategies **(B)** and synergistic combinations **(C)** are based on preclinical evidence, primarily from solid tumors; lymphoma-specific data are currently limited to cell-line and xenograft models. Solid boxes indicate strategies with published lymphoma data; dashed boxes indicate approaches extrapolated from other cancer types.

Inhibition of circRNA lowers the threshold for T-cell activation and also prevents the development of compensatory resistance (102). Evidence from preclinical studies also supports the existence of this synergistic effect. In esophageal squamous cell carcinoma, the combination of circNF1 knockdown with anti-PD-1 therapy has demonstrated a synergistic effect (93), whether this strategy is effective in lymphoma has not yet been tested (**Figure 3**).

## 7 Challenges, controversies, and future directions

We categorize the current knowledge on circRNA-mediated immune checkpoint regulation in lymphoma into three tiers: (1) Established mechanisms validated in lymphoma. These are few but well-characterized. The circPCBP2-miR-33a/b-PD-L1 axis in non-GCB DLBCL (57) represents the most thoroughly validated example, with functional knockdown and luciferase reporter assays confirming direct sponge activity. CircADARB1 in NKTCL (89) and viral circBARTs in EBV+ lymphomas (84) also fall into this category, though mechanistic depth varies. The circSCORE prognostic model in MCL (88) is clinically validated across independent cohorts. (2) Emerging evidence inferred from other cancer types. Many mechanistic concepts discussed in this review including circRNA-RBP interactions, micropeptide translation, and exosomal transfer have been experimentally demonstrated in solid tumors (e.g., melanoma, lung, breast cancer) but have not yet been confirmed in lymphoma. Throughout this review, we have explicitly indicated such extrapolations with phrases such as “although not yet reported in lymphoma”. (3) Open questions and hypotheses. Several ideas presented in the literature and discussed herein remain hypothetical: the “collective buffering” role of circRNA networks, the function of exosomal circRNAs as bona fide signaling molecules, the co-regulation of multiple checkpoints by a single circRNA, and the therapeutic potential of circRNA-derived neoantigens. These concepts are scientifically exciting but require dedicated experimental testing in lymphoma models. By explicitly distinguishing these three tiers, we aim to provide readers with a clear framework for assessing the strength of current evidence and identifying priority areas for future investigation.

The stoichiometric limitations of the ceRNA hypothesis remain a central controversy. We reiterate our recommendation that future studies report absolute copy numbers of circRNAs and miRNAs, along with their subcellular localization ratios, to enable rigorous stoichiometric assessment of ceRNA function. On a technological level, there are currently certain limits. For example, differentiating the roles of circRNAs from those of linear messenger RNAs remains a difficult task, and the efficacy of targeting techniques based on back-splicing junctions (BSJs) in primary cells has yet to reach an optimal level. Furthermore, the functional redundancy of circRNAs complicates the attribution analysis (103). Model system limitations must also be addressed, standard cell lines and xenograft models fail to adequately recreate the genetic variety, cellular heterogeneity, and dynamic tumor-immune interactions found in human lymphoma (104). As a result, developing genetically altered mice models with functional immune systems, as well as humanized patient-derived xenograft (PDX) models, is extremely valuable for study.

In the future, approaches such as single-cell and spatial transcriptomics may be used to better understand the regulatory networks of circRNAs in various cell types, as well as their spatial organization. To interpret the regulatory code, machine learning algorithms can be used to discover circRNAs that play a critical role (62). Synthetic biology methods can also be used to create circRNAs to encode tumor antigens or immunostimulatory molecules, allowing them to be used as therapeutic vaccines (105–107). Furthermore, combining circRNA regulation with CAR-T cell therapy can improve CAR-T cell survival by regulating circRNA networks. Integrating circRNAs profiling into clinical practice could enable dynamic monitoring of ICI responses and personalized combination therapies.

## 8 Conclusion

CircRNAs have risen from the background noise to become key regulators of gene expression. In lymphoma, they function as network hubs that integrate immunological, metabolic, and oncogenic signals to fine-tune immune checkpoint expression. The regulatory mechanisms are diverse ranging from miRNA sponging and RBP interactions to nuclear transcriptional control and micropeptide translation—and operate in both cell-intrinsic and cell-extrinsic compartments. The context-dependent nature of circRNA function underscores the need for subtype-specific studies and opens avenues for precision guided immunotherapy. Clinically, circulating circRNAs show promise as stable, dynamic biomarkers, which preclinical strategies including ASOs, CRISPR-Cas13, and nanodelivery offer potential therapeutic tools.

Crucially, the very controversies that surround the circRNA field—stoichiometric doubts about ceRNA, the low abundance of exosomal circRNAs, and the yet-unproven immunogenicity of micropeptides—should not be viewed as weaknesses, but rather as opportunities for breakthrough. We argue that it is precisely in the domains where current model systems fail and where quantitative discrepancies persist that circRNA research can make truly original advances. For instance, the ceRNA quantitative paradox forces us to move beyond the one-circRNA-one-miRNA linear model and embrace collaborative, subcellularly compartmentalized, and network-buffering mechanisms; the low absolute copy number of exosomal circRNAs compels us to think about signal amplification architectures, where rare triggers exploit intracellular feedback loops to produce large effects; and the yet-incomplete identification of circRNA-encoded micropeptides points to a largely unexplored proteome that may be especially rich in virally associated lymphomas. Therefore, future research should embrace these tensions as starting points for innovation—combining single-cell and spatial transcriptomics, stoichiometrically rigorous quantitation, and synthetic biology approaches to decode the regulatory logic of circRNAs. This review establishes circRNAs as multifaceted regulating elements at the junction of immunological, metabolic, and oncogenic signals, laying the groundwork for the development of next-generation circRNA-based immunotherapies to overcome resistance in lymphoma.
